# Supplementary material for: Single-cell radioluminescence microscopy with two-fold higher sensitivity using dual scintillator configuration
Source: PLoS One. 2020 Jul 7;15(7):e0221241. doi: 10.1371/journal.pone.0221241 (PMC7340323; doi:10.1371/journal.pone.0221241)
Supplement: Attachment — Submitted filename: Reviewer#2_response.pdf [file pone.0221241.s001.pdf]

Point-by-point response

The authors would like to thank the reviewers for their comments and suggestions that have helped improve the paper. Below, please find the point-by-point response to each comment:

Reviewer #2:

This study investigated an accelerated process to image human breast cancer cells MDA-MB-231 that was imaged with two scintillator slabs after incubated with [18F]FDG. Although the authors claimed to improve the sensitivity to 2-fold and cut the data acquisition time into half, I am not convinced that this signal increase is due to positron emission.

Q1: This study used two CdWO4 scintillators that sandwiched these MDA-MB-231 cancer cells. As each scintillator is 0.5 mm in thickness, therefore together they have 1 mm thickness, that would have reduced intrinsic efficiency as low as 10% due to anhelation photons. Then how this is possible to have sensitivity increased by 2-fold specifically coming from positron emission.

Author response: The authors agree that the intrinsic efficiency will be ~ 10% for 511 keV annihilation photons. However, the principle of radioluminescence microscopy is to capture the scintillation signals from positrons instead of the 511 keV photons. Since the average energy of the beta particles from ^{18}F decay is ~ 250 keV, most of the energy is deposited close to the scintillator surface with an average penetration depth of 25 µm, which matches the optical depth of field of the microscope. In contrast, annihilation photons interact with the scintillator at various depths, mainly outside the microscope depth of focus, and the scintillation signal from these photons is typically unfocused and spread out, thus it is rejected by the data processing algorithm. see below a figure from our previous publication that illustrates the differences in scintillation signal between a beta track and an annihilation photon.

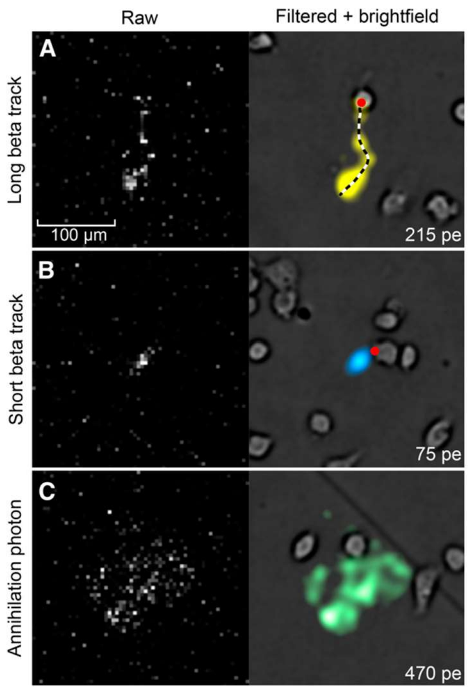

Figure. Examples of ionization tracks. (a) Long ionization track, originating from cell. Dashed line is estimated positron trajectory. (b) Shorter ionization track, likely produced by low-energy positron or positron traveling orthogonally to imaging plane. Red dot is estimated decay location. (c) Annihilation photon interacting deep in scintillator and producing diffuse, unfocused signal. Such signals are not used in final image. Number of photoelectrons (pe) recorded by CCD sensor for each event is indicated. Color is shown for illustration purpose.

Referenced from Guillem Pratx et. al, “High-Resolution Radioluminescence Microscopy of 18F-FDG Uptake by Reconstructing the β-Ionization Track,” 2013, Journal of Nuclear Medicine, doi: 10.2967/jnumed.112.113365.

Due to the 1) short penetration distance of positrons and 2) the scintillators covering the top and bottom of the radioactive source (e.g. radiolabeled cells; please see Figure 1), the authors believe that the proposed approach significantly enhances the detection sensitivity.

Q2: The authors did not address this issue how they account for these photons in the Monte Carlo simulation.

Author response: The positron-electron annihilation was included in the simulation model. However, as the annihilation event happened at the last step of positron transport/deposition in the scintillator, only two 511 keV photons are produced. Of the two 511 keV photons, only one may reach the imaging area, which is quite a small number relative to the cascade of optical photons produced due the positron’s interaction with the scintillator. In conjunction with the reason mention in Q1, the 511 keV photons are negligible even if they are detected.

The following phrase was added in the caption under Fig 5 to indicate the contribution of annihilation photons:

"The scintillation from 511 keV photons, which are photons produced from electron-positron annihilation events, were also included in the simulation but were negligible since they have a smaller probability than positrons to deposit energy within the ~25 µm-thick region of the scintillator that is in focus in the microscope."

Also, the authors felt that more information regarding the Monte Carlo simulation is needed, and the following phrase was added in the Materials and Methods section to further clarify the procedure:

"An optical model was implemented by convoluting the simulated scintillation tracks with the 3D point-spread function of the microscope's objective, then applying pixel noise characteristic of the EMCCD camera. Radioluminescence images were then reconstructed by selecting and counting the scintillation events occurring from the beta particles."

Q3: Also, in the Monte Carlo Simulation source was positioned 5 µm above a 100 um thick CdWO4 slab. It is not clear from the why this space was added. It may be beneficial to clarify this aspect.

Author response: The authors realized that there was no explanation in the manuscript to explain the reasoning behind this offset. The 5 µm offset corresponds to the center of a single cancer cell with an approximate height of 10 µm. While the difference is large between different cell types, previously results indicate that this height is an appropriate simulating MDA-MB-231 cells (Qian Wang et. al, "Performance evaluation of 18F radioluminescence microscopy using computational simulation," 2017, Medical Physics, doi: doi.org/10.1002/mp.12198).

The following phrase was added in the Materials and Methods section to explain about the offset:

"The 5 µm offset corresponds to the height of the MDA-MB-231 cells assuming an average height of 10 µm, as previously observed [19]."

Q4: the study is interesting, Also, the study is failed to show clinical pull for this imaging technique. It will be helpful to add a new paragraph in the Introduction section.

Author response: The authors appreciate the suggestion of potentially applying the technique in the clinical space. The following phrase has been added in the Introduction:

"Since the time required to acquire radioluminescence images can be considerably reduced, potential applications of the technique include identifying radiolabeled histological tumor samples from patients and screening the efficacy of novel radiopharmaceuticals in biological specimens."
